# Supplementary material for: Microbial Biosynthesis of Chrysazin Derivatives in Recombinant Escherichia coli and Their Biological Activities
Source: Molecules. 2022 Aug 29;27(17):5554. doi: 10.3390/molecules27175554 (PMC9457698; doi:10.3390/molecules27175554)
Supplement: Supplementary file 1 [file molecules-27-05554-s001.zip › molecules-1853046-supplementary.pdf]

Supplementary data of

Microbial Biosynthesis of chrysazin derivatives in Recombinant *Escherichia coli* and their biological activities

Purna Bahadur Poudel[1*], Dipesh Dhakal[1*], Rubin Thapa Magar[1], and Jae Kyung Sohng[1,2,]**

[1]*Institute of Biomolecule Reconstruction (iBR), Department of Life Science and Biochemical Engineering, Sun Moon University, 70 Sun Moon-ro 221, Tangjeong-myeon, Asan-si, Chungnam 31460, Korea.*

[2]*Department of Biotechnology and Pharmaceutical Engineering, Sun Moon University, 70 Sun Moon-ro 221, Tangjeong-myeon, Asan-si, Chungnam 31460, Korea.*

[*]These authors equally contributed.

**Corresponding author: Prof. Jae Kyung Sohng

Tel: +82 (41) 530-2246

Fax: +82 (41) 530-8229

Email: sohng@sunmoon.ac.kr

Table and figure of contents

Table S1. Anticancer (IC50 (µM)) potential of chrysazin analogues against different cell lines.

Cell Lines	Chrysazin (Chy)	CR	CRM
**Cancer cell lines**			
**AGS**	17.08	28.58	7.513
**Huh7**	30.53	21.28	4.467
**HL60**	22.24	14.68	4.540
**Normal cell lines**			
**HaCaT**	>200	>200	>200

Table S2. Antibacterial activities test against Gram-positive and Gram-negative bacteria via disc diffusion assay. The zone of inhibition (diameter) due to DMSO, chrysazin, chrysazin-8-*O*-*α*-L-rhamnoside, and chrysazin-8-*O*-α-L-2′-*O*-methylrhamnoside against different pathogens is noted in mm.

	DMSO	Chrysazin	CR	CRM
**Gram-Positive Strains**				
S. aureus CCARM 3090 (MRSA)	ND	7 ± 0.25	11 ± 0.25	14 ± 0.25
S. aureus CCARM 3640 (MRSA)	ND	ND	12 ± 0.27	15 ± 0.38
S. aureus CCARM 3089 (MRSA)	ND	ND	7 ± 0.23	10 ± 0.25
S. aureus CCARM 33591(MRSA)	ND	ND	ND	9 ± 0.45
S. aureus CCARM 0205 (MSSA)	ND	ND	15 ± 0.15	20 ± 0.35
S. aureus CCARM 0204 (MSSA)	ND	ND	11 ± 0.35	16 ± 0.27
S. aureus CCARM 0027 (MSSA)	ND	ND	8 ± 0.11	11 ± 0.25
S. aureus CCARM 3634 (MRSA)	ND	ND	12 ± 0.15	14 ± 0.28
S. aureus CCARM 3635 (MRSA)	ND	ND	7 ± 0.17	10 ± 0.16
Bacillus subtilis ATCC 6633	ND	ND	ND	ND
Enterococcus faecalis 19433	ND	ND	ND	ND
Enterococcus faecalis 19434	ND	ND	ND	ND
Kocuria rhizophilla NBRC 12708	ND	ND	ND	ND
Micrococcus luteus	ND	ND	ND	ND
**Gram-Negativ Strains**				
Escherichia coli ATCC 25922	ND	ND	ND	ND
Proteus hauseri NBRC3851	ND	ND	ND	ND
Klebsiella pneumonia ATCC10031	ND	ND	ND	ND
Salmonella enterica ATCC 14028	ND	ND	ND	ND

ND: not detected.

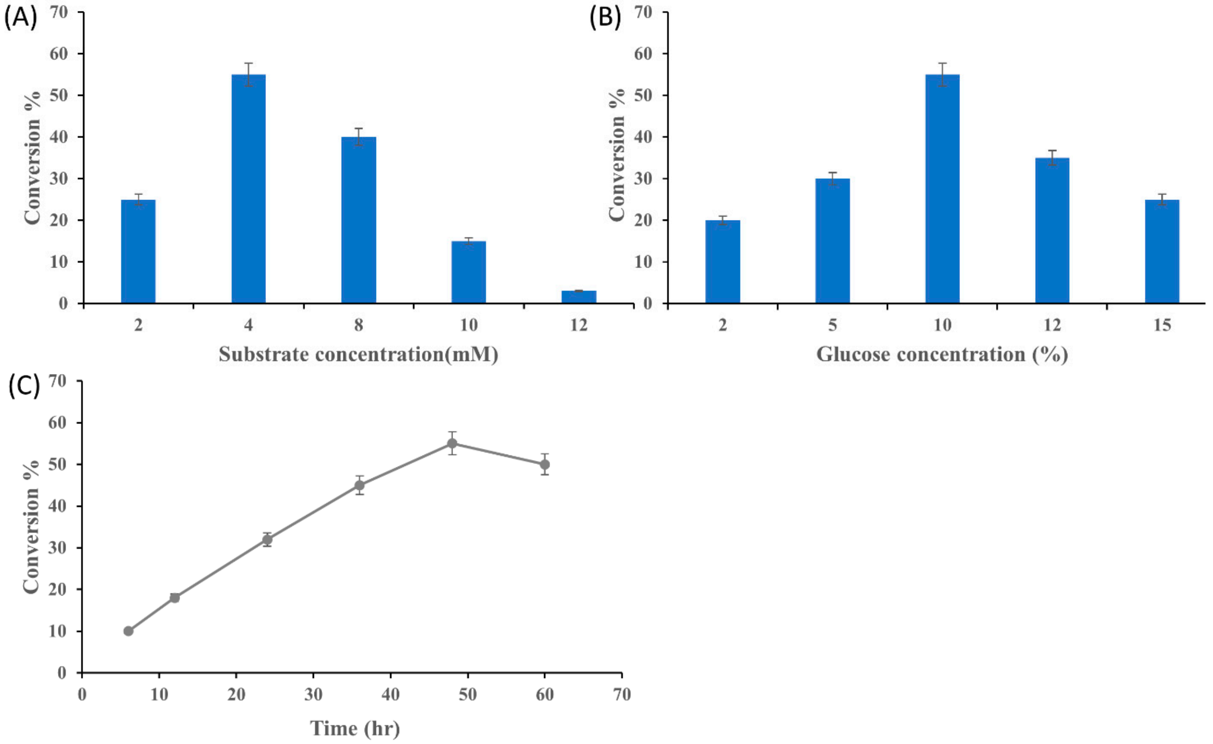

Figure S1.

Determination of optimum culture conditions using chrysazin as substrate. (**A**)The conversion rate of chrysazin using different substrate concentrations; the optimum concentration is 4 mM. (**B**) Conversion rate using different concentrations of glucose with the already determined substrate condition; the optimum glucose concentrations 10%. (**C**) Conversion rate of chrysazin in different time interval with the already determined conditions; the optimal time is 48 hrs. (Error bars show the standard deviation of three distinct experiments).

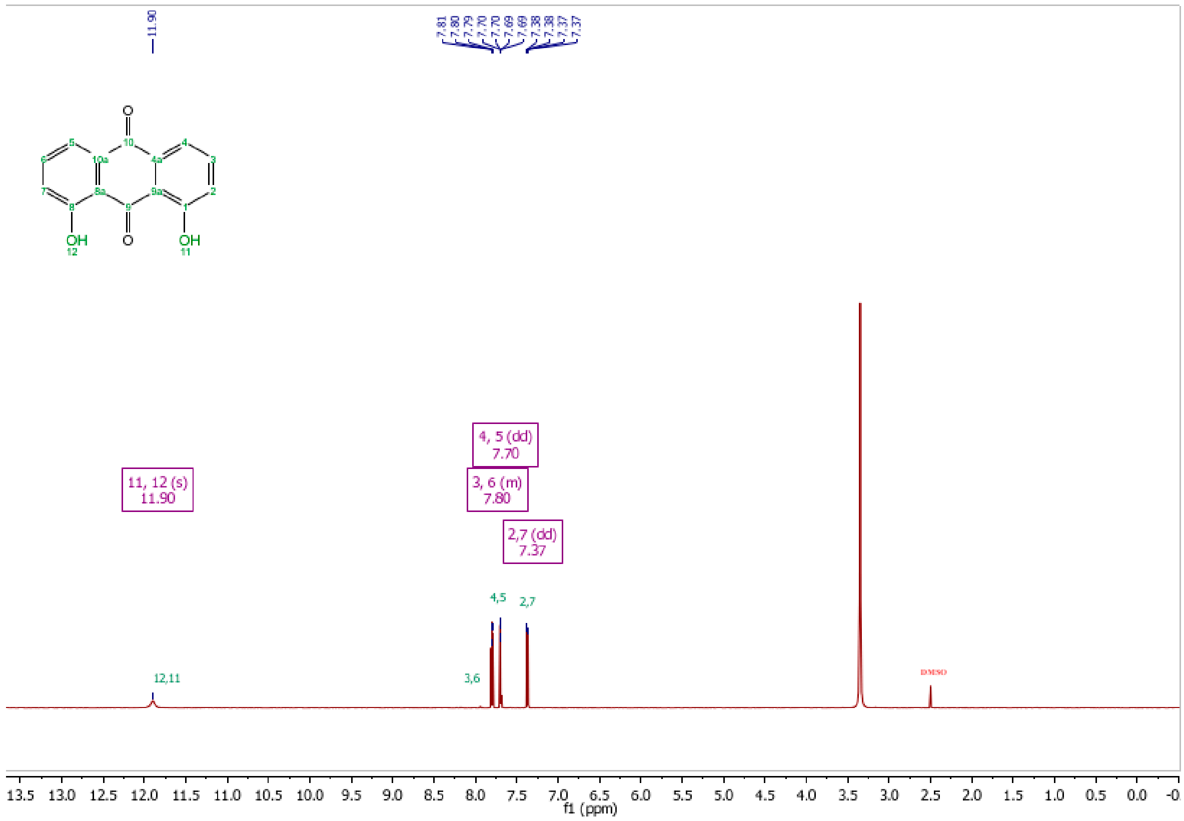

Figure S2.

(a) ^{1}H NMR spectrum of chrysazin at 300MHz in DMSO-*d6*

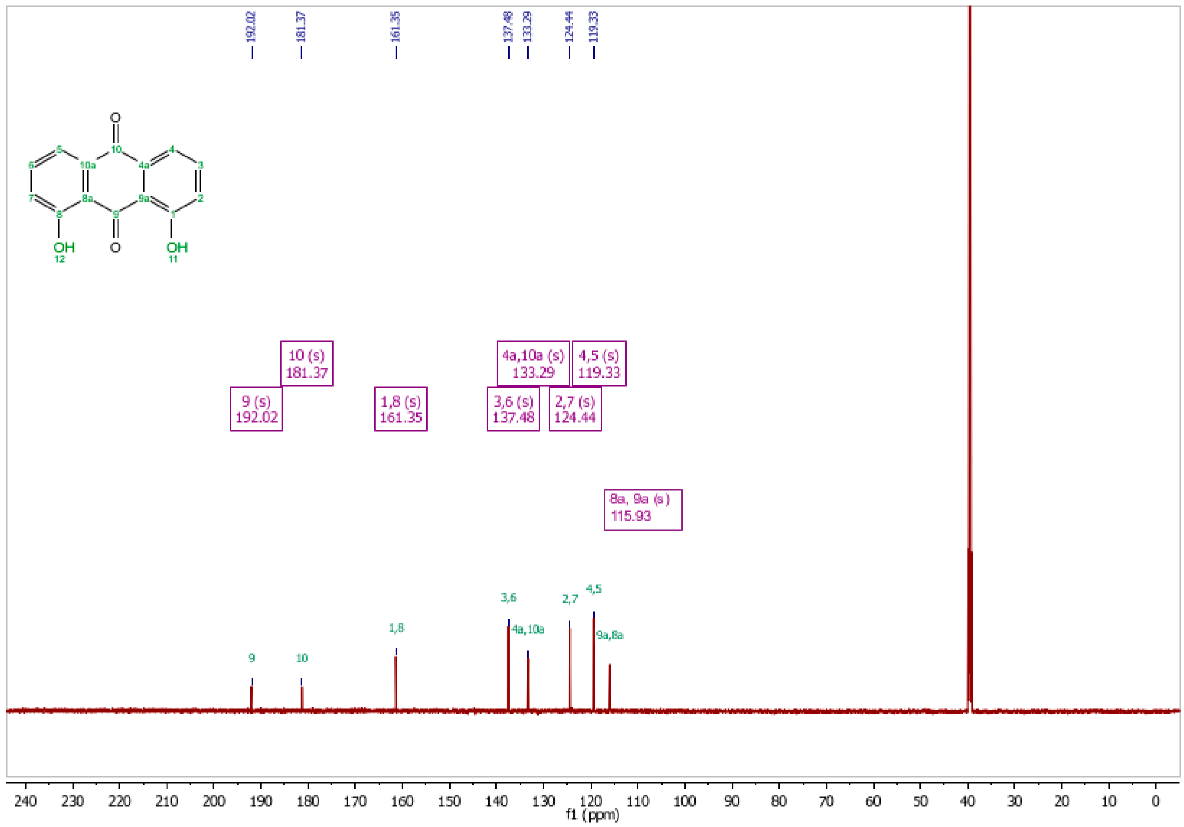

(b) ^{13}C NMR spectrum of chrysazin 176 MHz in DMSO-*d6*

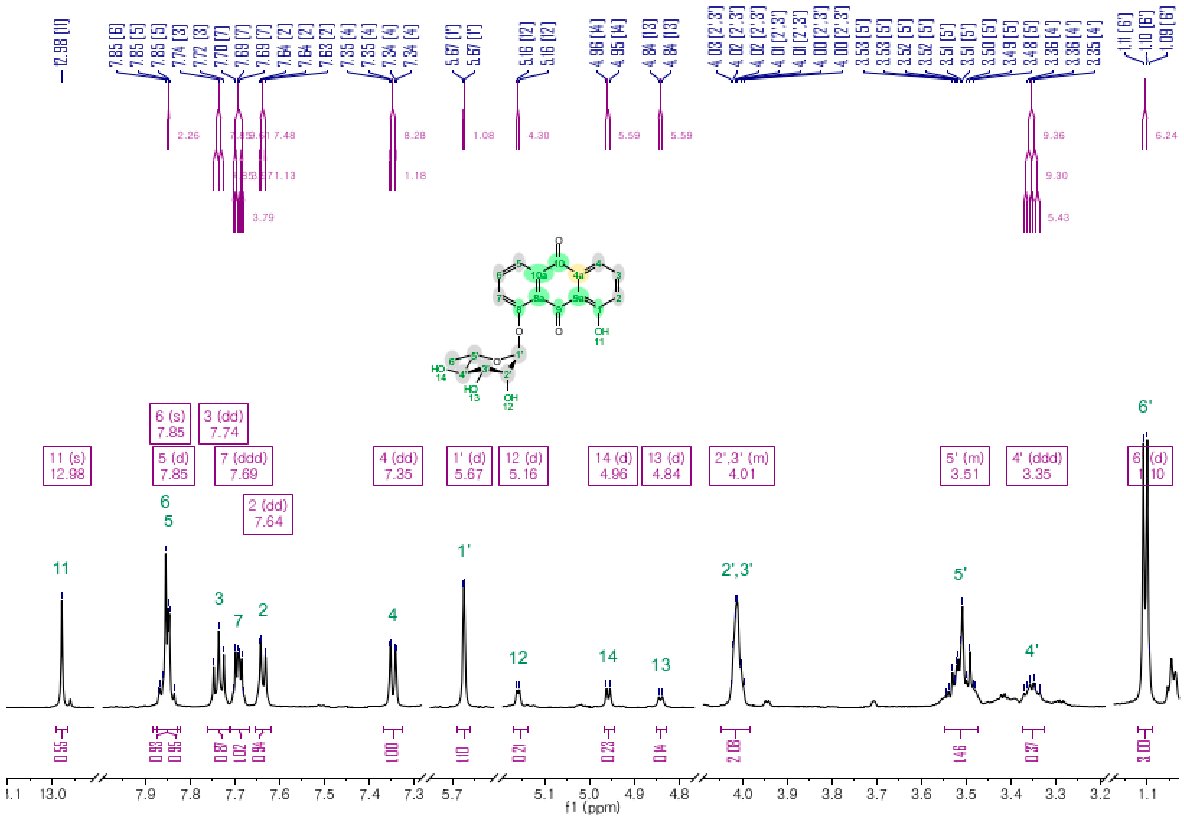

Figure S3. Different NMR spectrum of chrysazin-8-*O*-*α*-L-rhamnoside

(a) ^{1}H NMR spectrum of chrysazin-8-*O*-*α*-L-rhamnoside in 700MHz, DMSO-*d6*

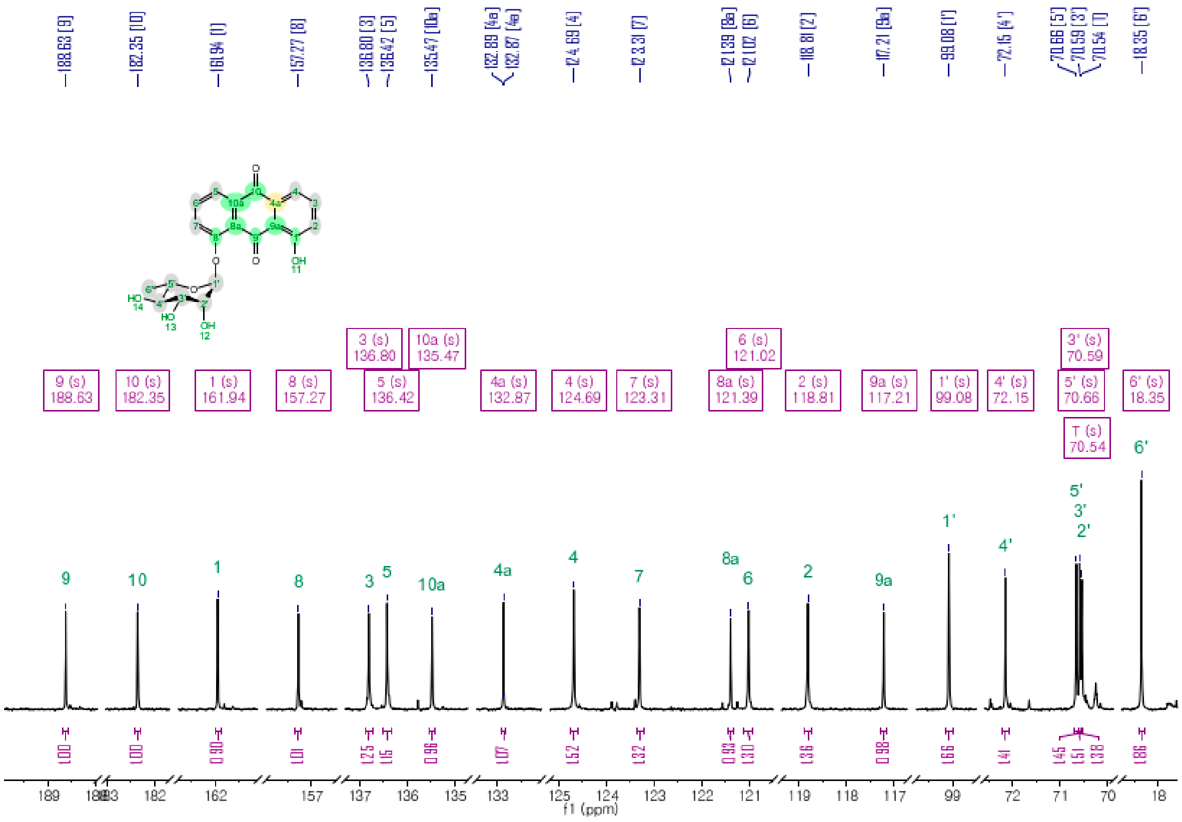

(b) ^{13}C NMR spectrum of chrysazin-8-*O*-*α*-L-rhamnoside in 176 MHz, DMSO-*d6*

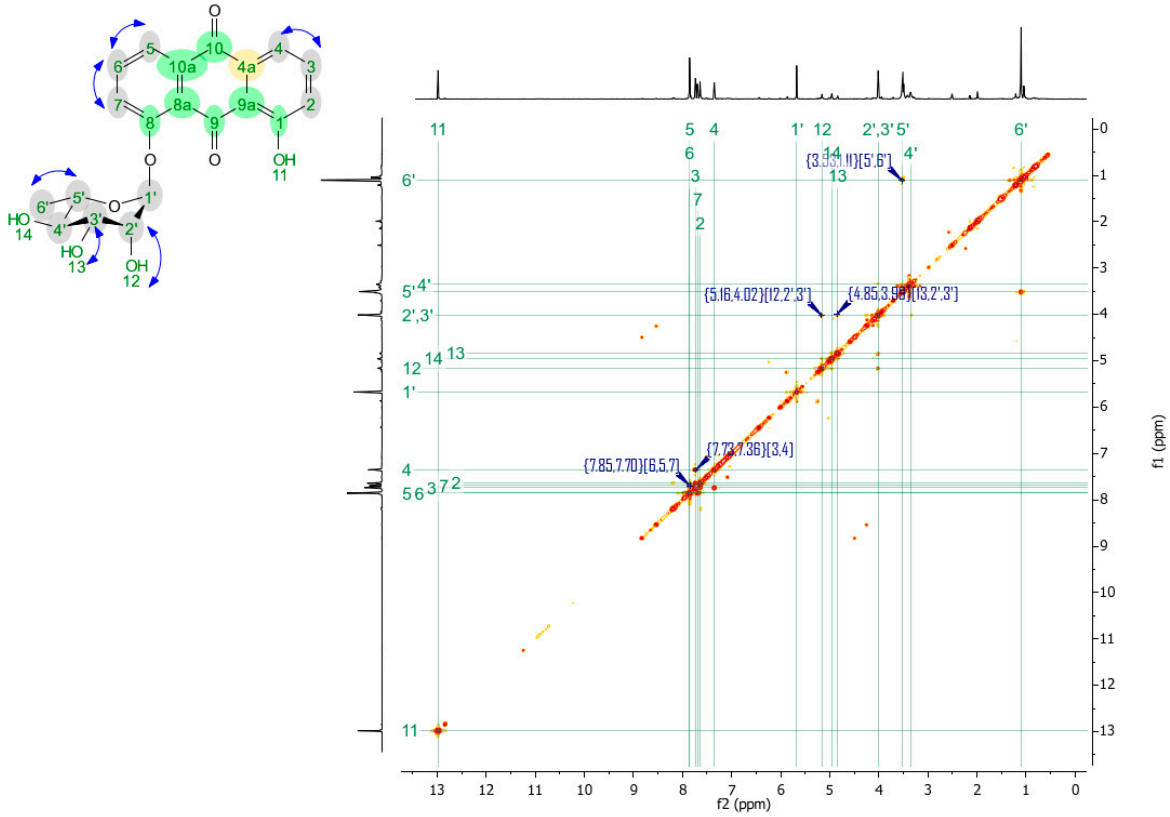

(c) ^{1}H-^{1}H COSY NMR of chrysazin-8-*O*-*α*-L-rhamnoside

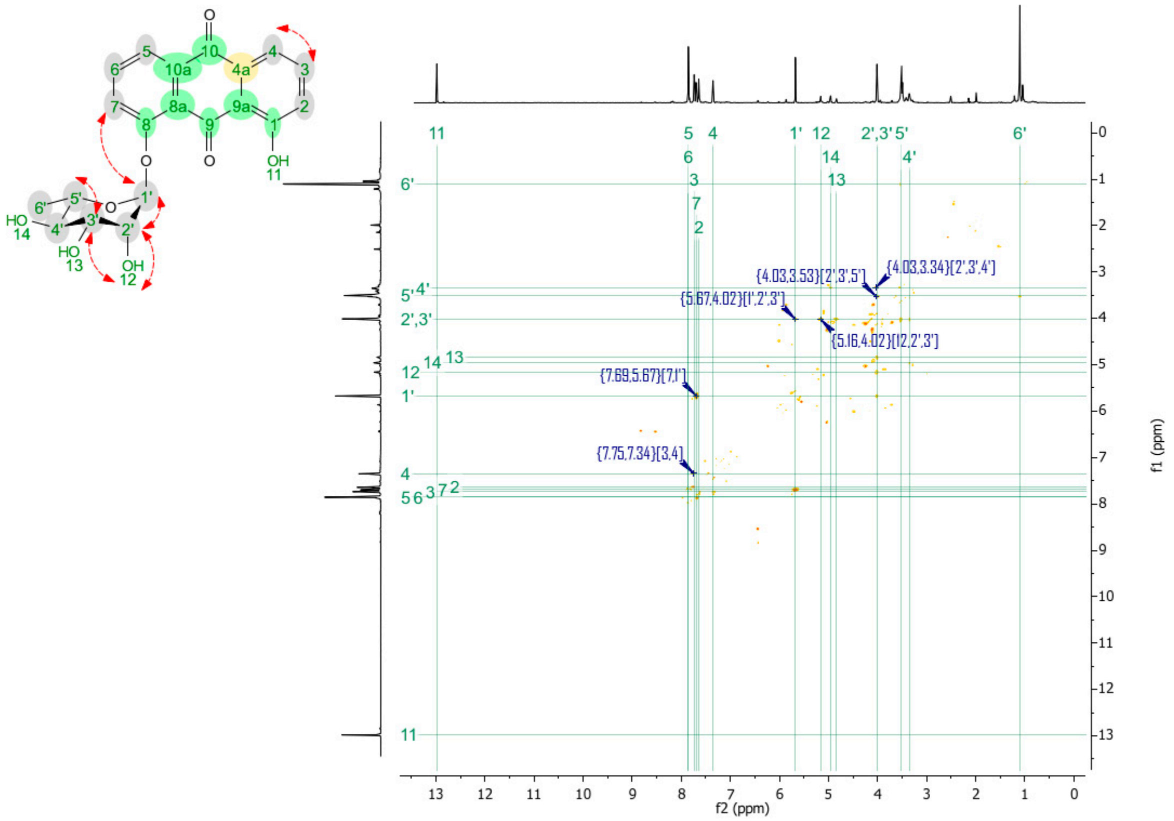

(d) ¹H-¹H ROSEY NMR of chrysazin-8-*O*-*α*-L-rhamnoside

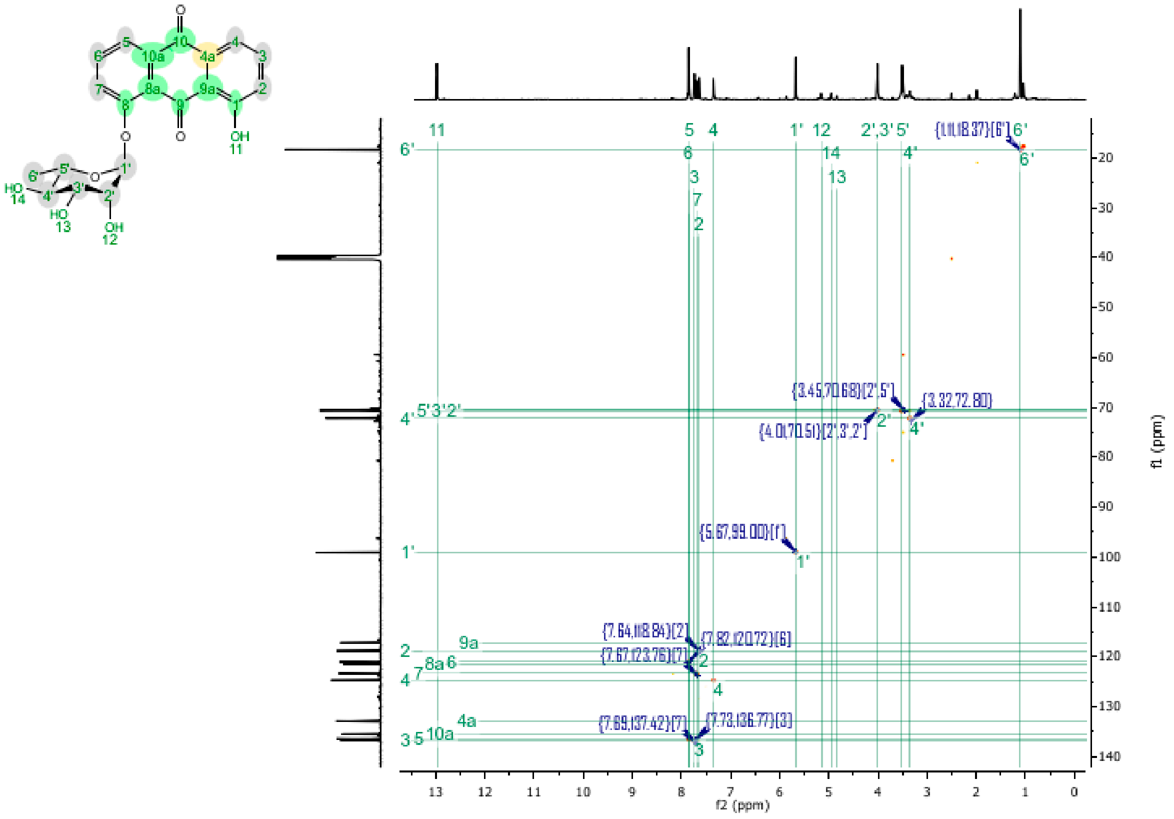

(e) ^{1}H-^{13}C HSQC-DEPT NMR of chrysazin-8-*O*-*α*-L-rhamnoside

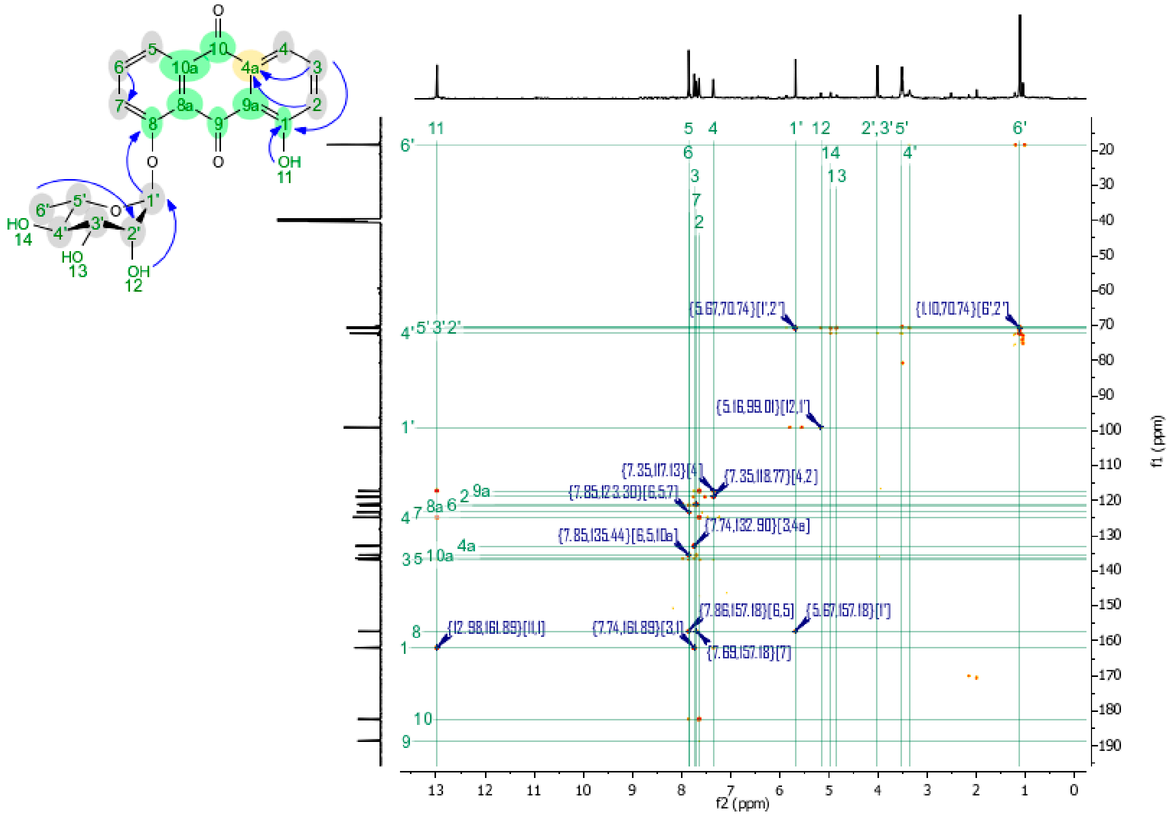

(f) ^{1}H-^{13}C HMBC NMR of chrysazin-8-*O*-*α*-L-rhamnoside

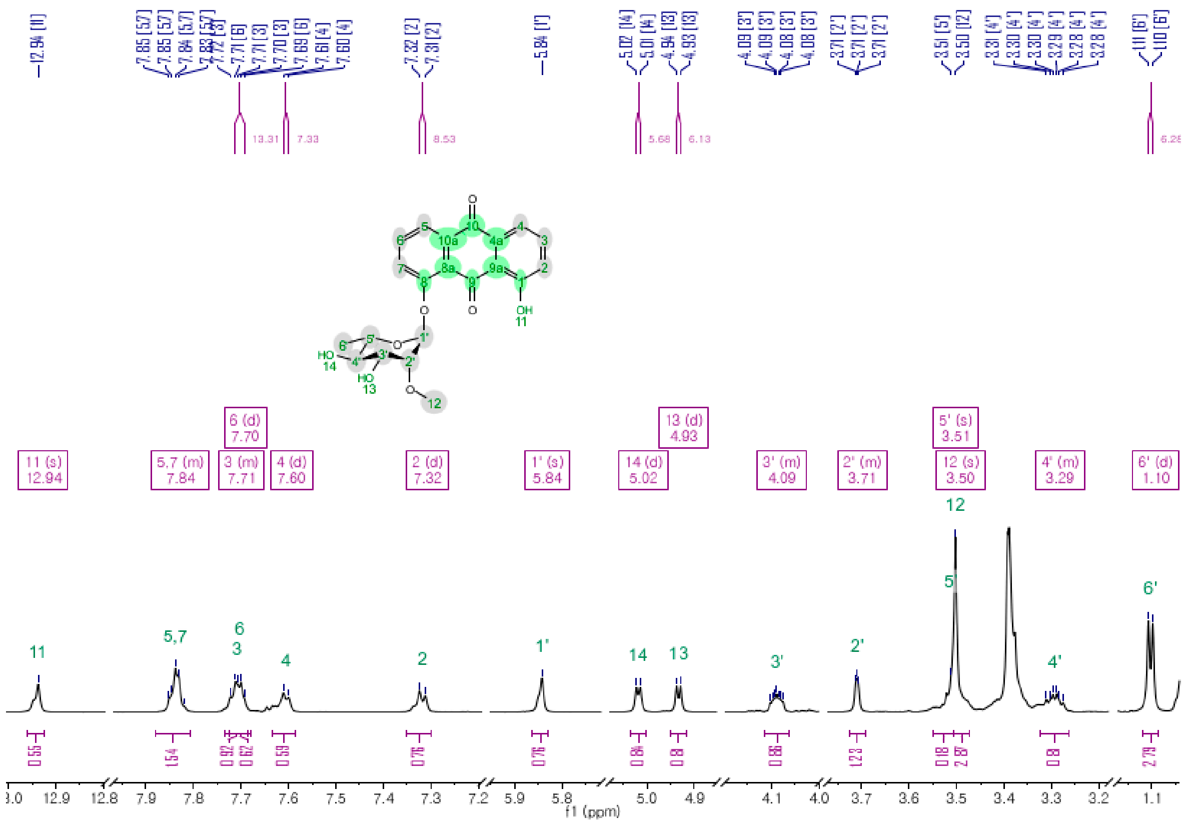

Figure S4. Different NMR spectrum of chrysazin-8-*O*-α-L-2′-*O*-methylrhamnoside

(a) ^{1}H NMR spectrum of chrysazin-8-*O*-α-L-2′-*O*-methylrhamnoside at 700MHz in DMSO-*d6*

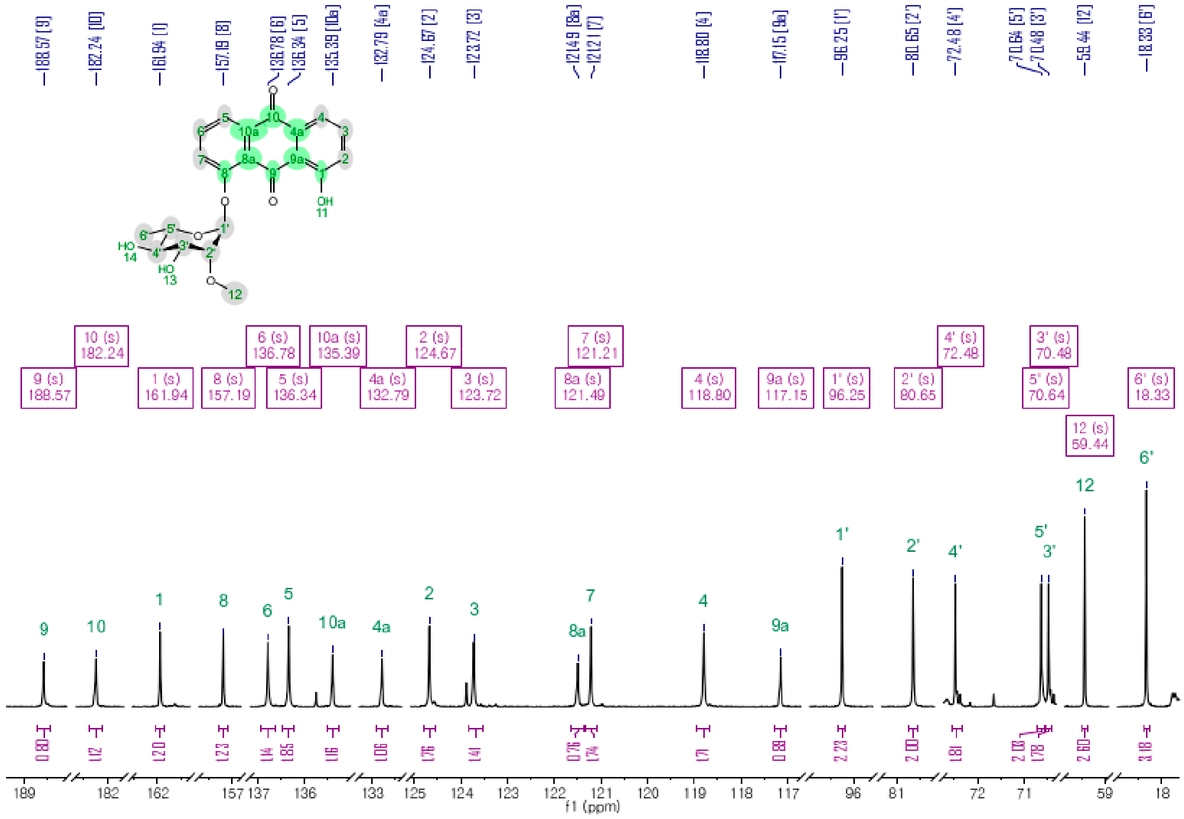

(b) ^{13}C NMR spectrum of chrysazin-8-*O*-α-L-2′-*O*-methylrhamnoside in 176 MHz, DMSO-*d6*

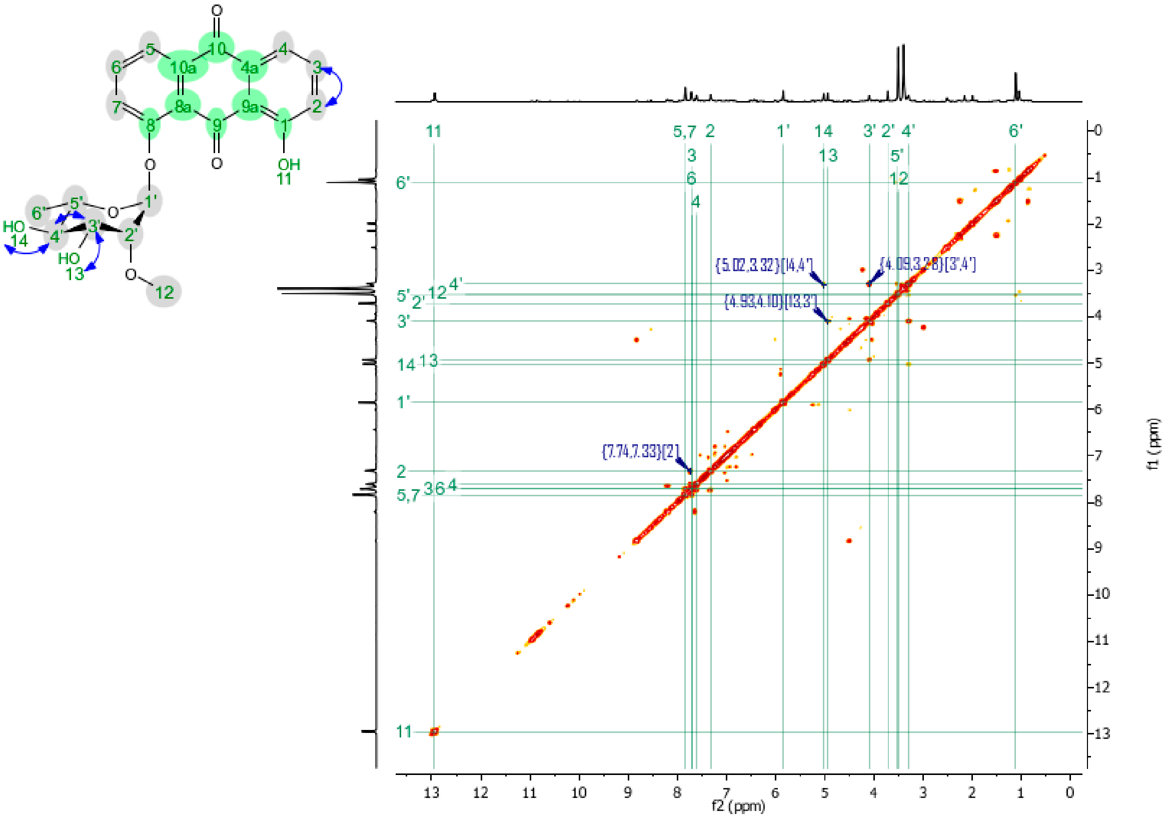

(c) ^1H-^1H COSY NMR of chrysazin-8-*O*-α-L-2′-*O*-methylrhamnoside

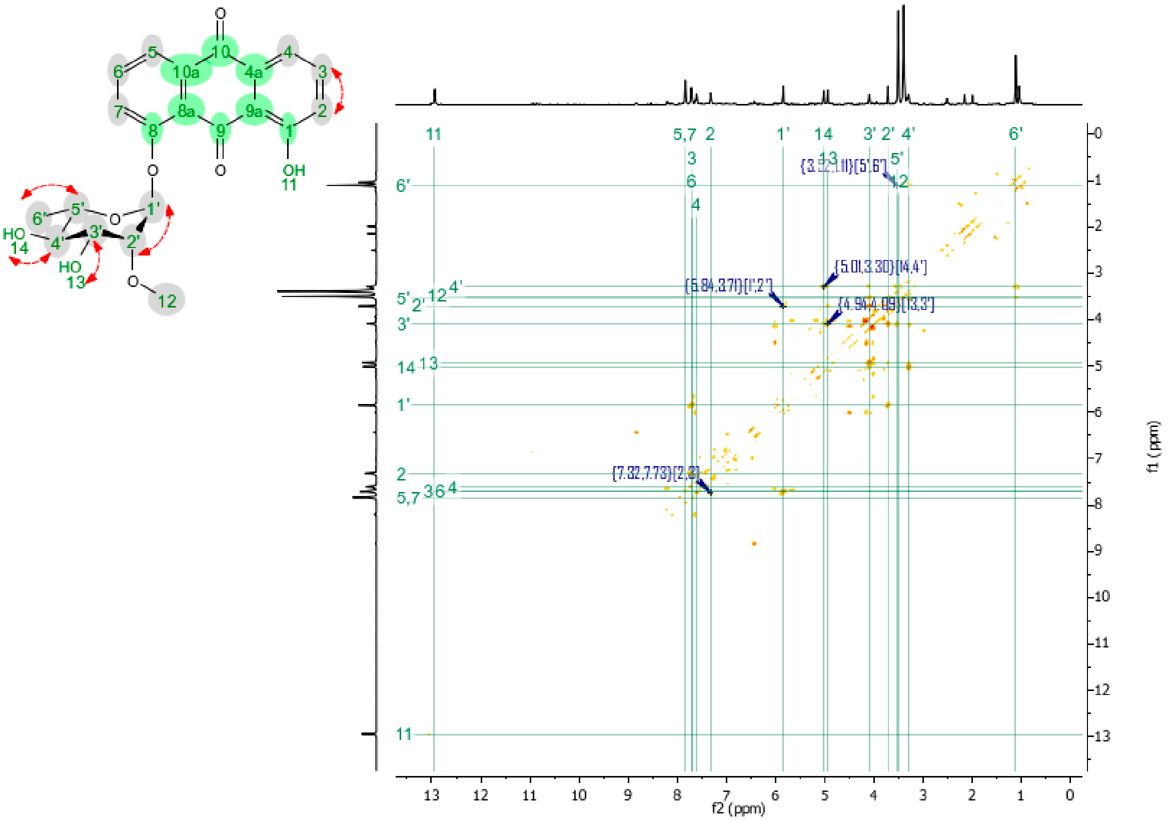

(d) ^{1}H-^{1}H ROSEY NMR of chrysazin-8-*O*-α-L-2′-*O*-methylrhamnoside

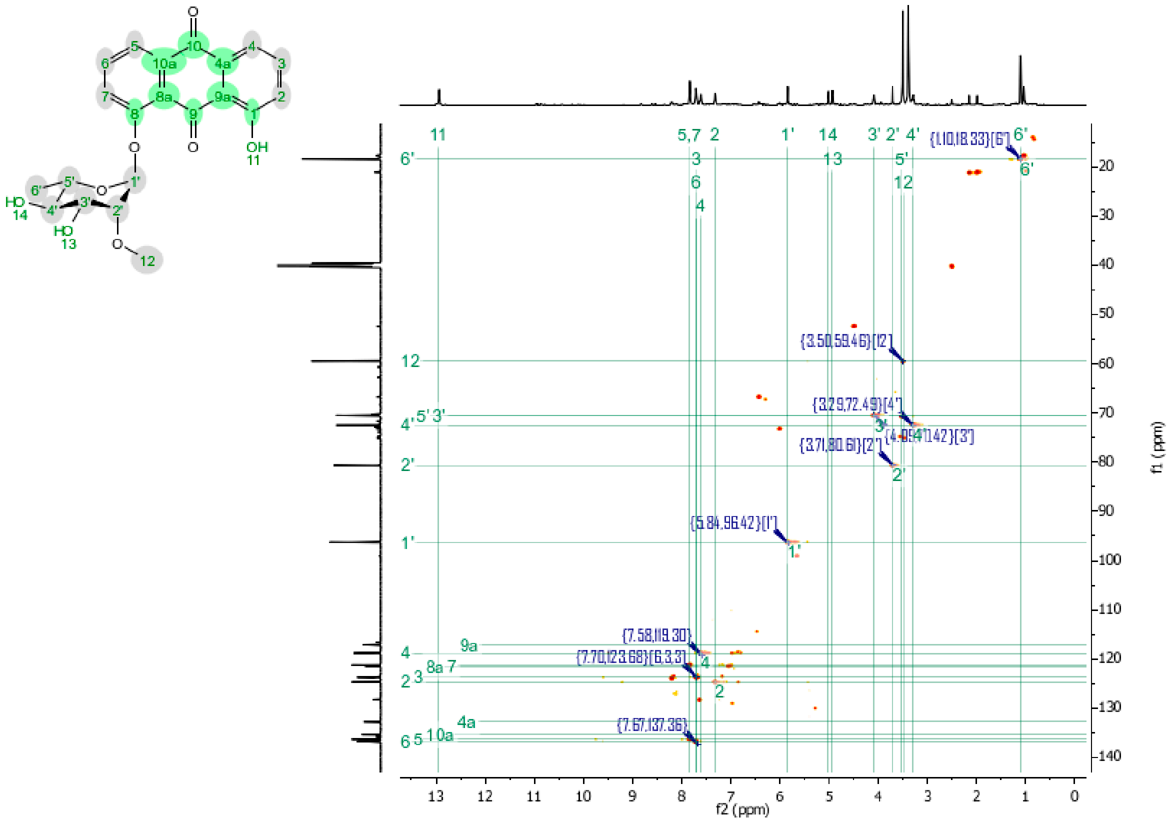

(e) ^{1}H-^{13}C HSQC-DEPT NMR of chrysazin-8-*O*-α-L-2′-*O*-methylrhamnoside

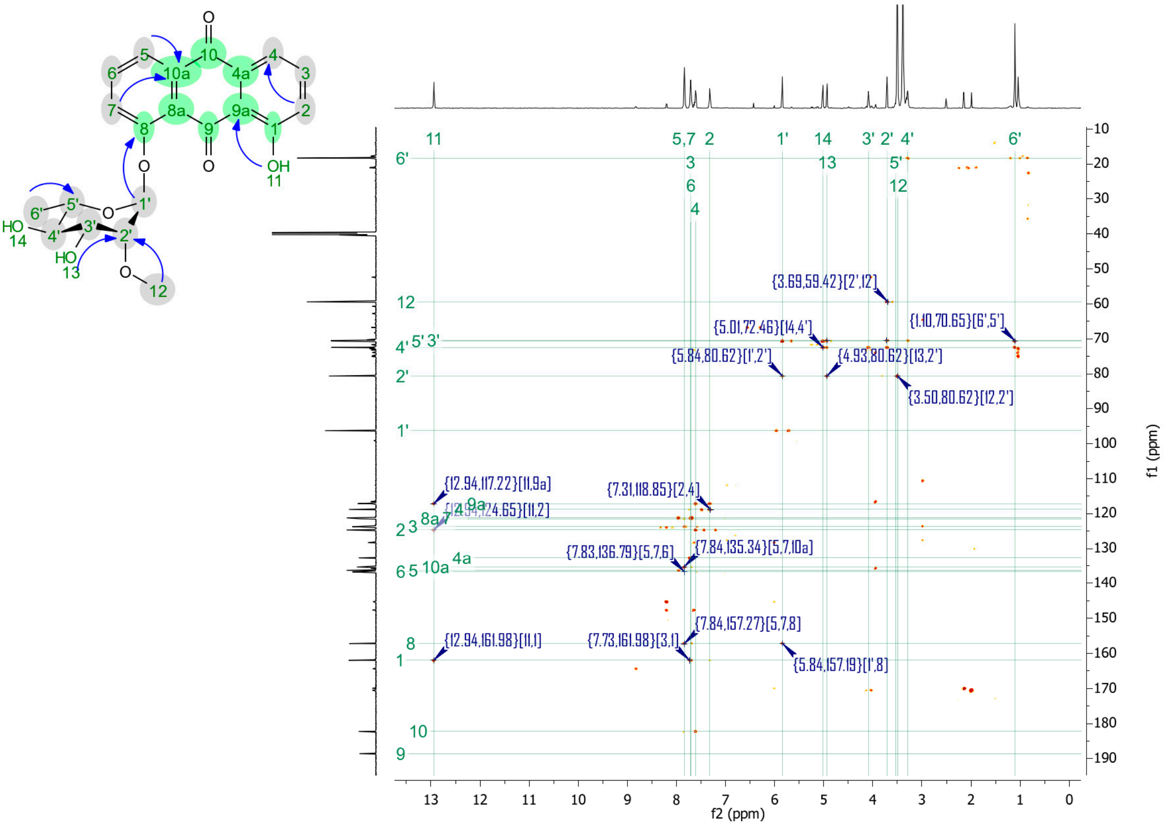

(f) ^{1}H-^{13}C HMBC NMR of chrysazin-8-*O*-α-L-2′-*O*-methylrhamnoside

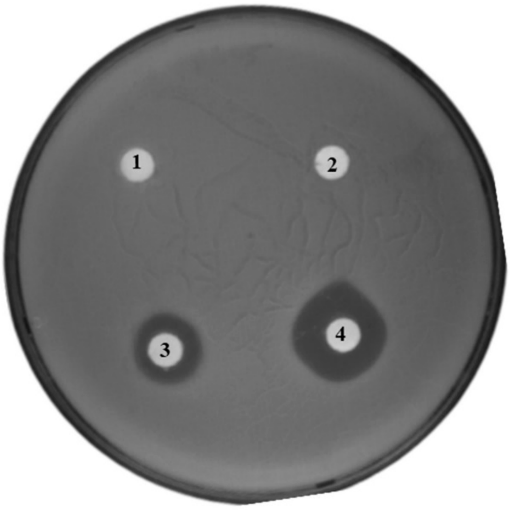

(i)

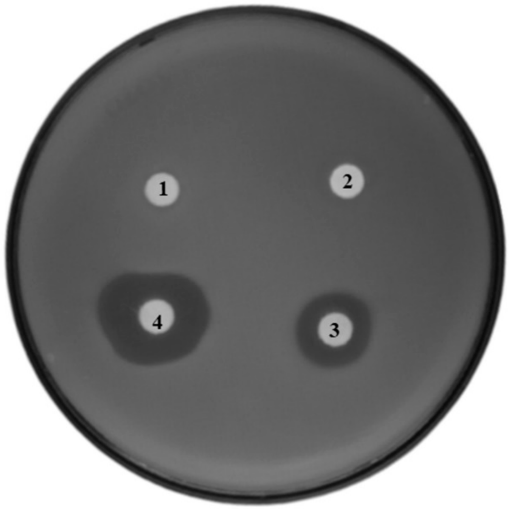

(ii)

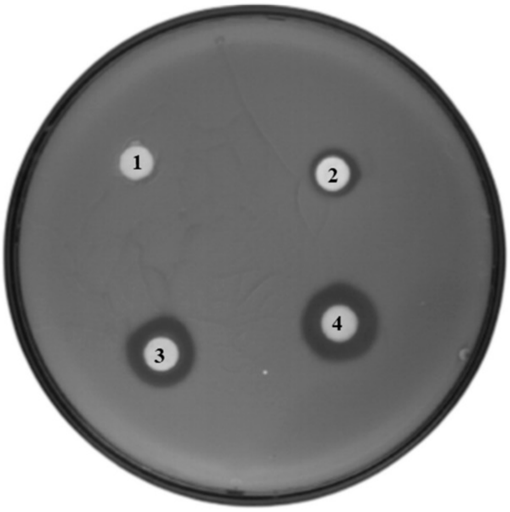

(iii)

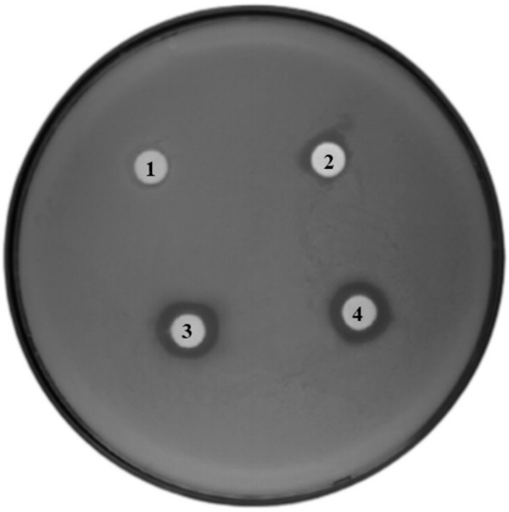

(iv)

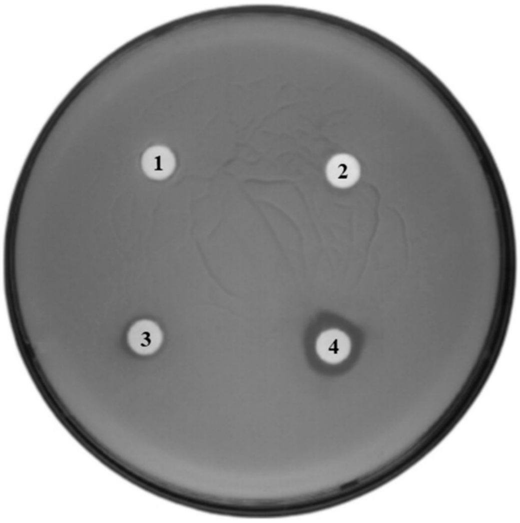

(v)

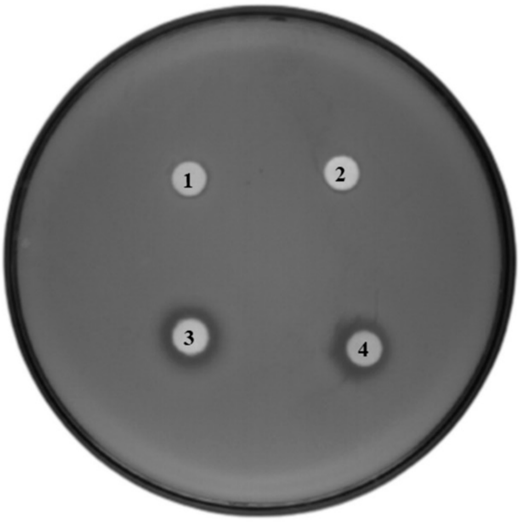

(vi)

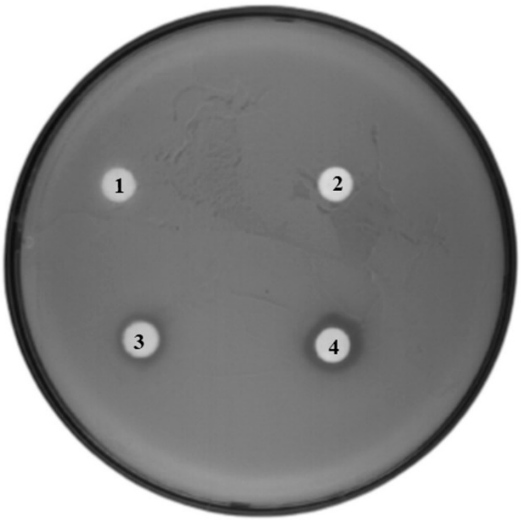

(vii)

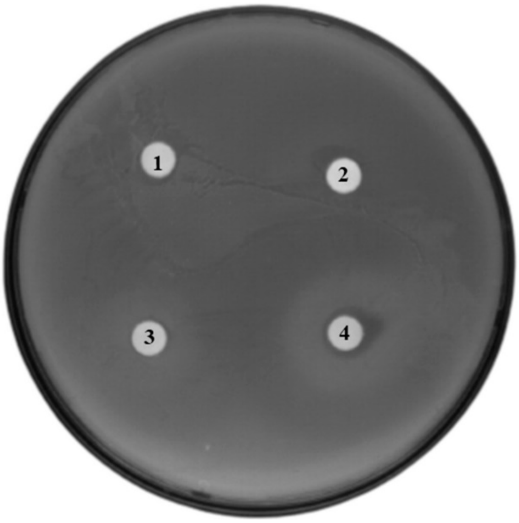

(viii)

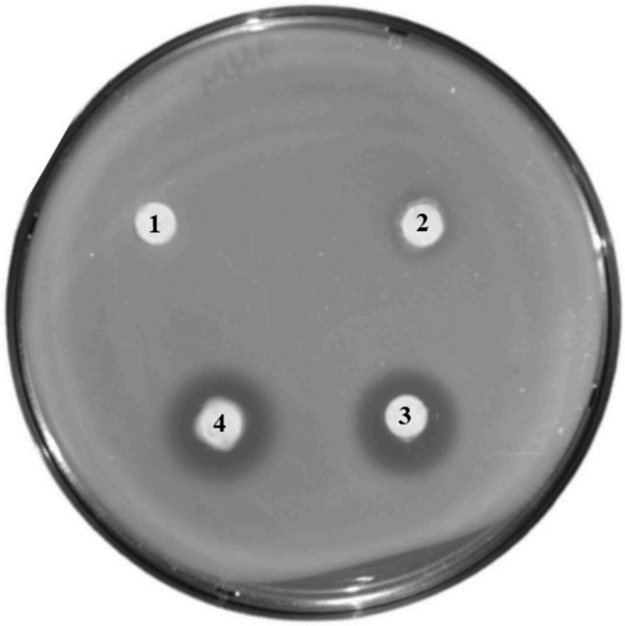

(ix)

Figure S5. Antibacterial activity of 1(DMSO), 2(Chrysazin), 3(CR), and 4(CRM) with (i) *S. aureus* CCARM 0204 (MSSA), (ii) *S. aureus* CCARM 0205 (MSSA), (iii) *S. aureus* CCARM 3090 (MRSA), (iv) *S. aureus* CCARM 3634 (MRSA), (v) *S. aureus* CCARM 3635 (MRSA), (vi) *S. aureus* CCARM 0027 (MSSA), (vii) *S. aureus* CCARM 3089 (MRSA), (viii) *S. aureus* CCARM 33591(MRSA) (ix) *S. aureus* CCARM 3640 (MRSA).

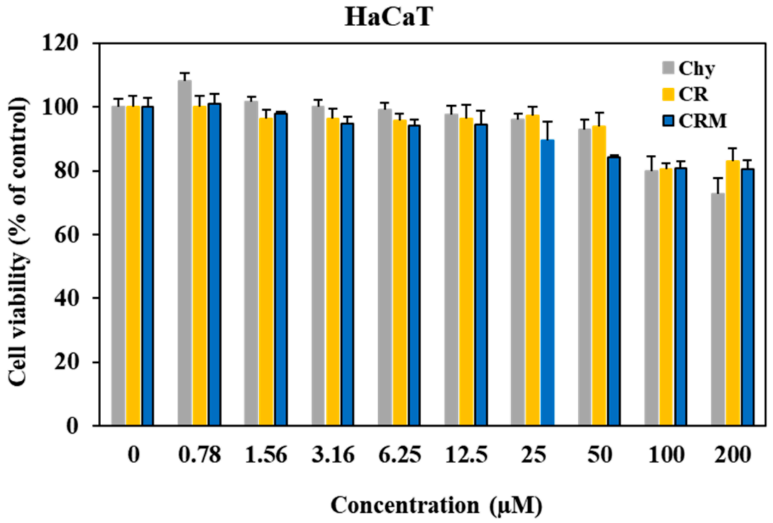

Figure S6. Inhibitory effects of chrysazin, CR, and CRM on normal cell HaCaT growth.
